# Validation of the Kidney Failure Risk Equation in the Colombian Population

**DOI:** 10.1155/2024/1282664

**Published:** 2024-02-17

**Authors:** C. Larrarte, J. Vesga, F. Ardila, A. Aldana, D. Perea, M. Sanabria

**Affiliations:** ^1^Baxter Renal Care Services, Bucaramanga, Colombia; ^2^Baxter Renal Care Services-Latin America, Bogotá, Colombia; ^3^Baxter Renal Care Services, Bogota, Colombia

## Abstract

**Introduction:**

Chronic kidney disease prevention programs must identify patients at risk of early progression to provide better treatment and prolong kidney replacement therapy-free survival. Risk equations have been developed and validated in cohorts outside of Colombia, so this study aims to evaluate the discrimination and calibration of the four-variable kidney failure risk equation in a Colombian population where it has yet to be validated.

**Methods:**

External validation study of a kidney failure risk equation using a historical cohort of patients with CKD stages 3, 4, and 5, adults without a history of dialysis or kidney transplantation with a two-year follow-up, belonging to the Baxter Renal Care Services Colombia network. The discriminatory capacity of the model was evaluated by the concordance index using Harrell's C statistic, and the time-dependent area under the receiver operating characteristic (ROC) curve was estimated using the nearest neighbor method, as well as the optimal cut-off point for sensitivity and specificity. Calibration was determined by the degree of agreement between the observed outcome and the probabilities predicted by the model using the Hosmer–Lemeshow statistic.

**Results:**

A total of 5,477 patients were included, with a mean age of 72 years, 36.4% diabetic, and a mean baseline eGFR of 36 ml/min/1.73 m^2^. The rate of dialysis initiation was three events per 100 patient-years, 95% CI (2.9–3.6). The optimal cutoff for sensitivity was 0.94, for specificity, 0.76, and the area under the ROC curve was 0.92. Harrell's C-statistic was 0.88 for the total population, 0.88 for diabetic patients, and 0.93 for those 65 years or older. The validation of the model showed good calibration.

**Conclusions:**

In this Colombian cohort, the four-variable KFRE with a two-year prediction horizon has excellent calibration and discrimination, and its use in the care of CKD Colombian patients is recommended.

## 1. Introduction

Chronic kidney disease (CKD) is a syndrome with multiple pathophysiological features that have been elucidated for decades [1–4]. It is a highly prevalent disease associated with a declining quality of life, morbidity, and higher mortality rates [5, 6]. One of the cornerstones of CKD treatment is based on measures that aim to halt the deterioration of kidney function, prevent the need for dialysis, and reduce the risk of cardiovascular events [7, 8]. Considering that the progression of CKD increases the burden of disease and the cost of care, it is essential to understand the risk of progression to establish preventive or therapeutic measures that can positively influence patient outcomes.

Several demographic and clinical characteristics may be associated with differences in the rate of progression to kidney failure, defined as the need for dialysis or kidney transplant [9–13].

Furthermore, the development of clinical epidemiology has led to an understanding of how multiple risk factors can be combined to generate a prognostic risk model [14]. The use of these models in daily clinical practice has become a valuable tool for making prognostic and therapeutic decisions, although it is not without methodological limitations [15]. Among these, a critical step before the widespread use of a risk model in each jurisdiction is the external validation to assess its predictive performance in different patient populations and, when necessary, adapt the model to local circumstances or include new predictors [16, 17].

Specifically, in patients with CKD, some predictive models for progression to kidney failure were developed using inappropriate methods and were poorly reported [18]. Subsequently, Tangri et al. developed a predictive model for the progression of CKD to kidney failure in the Canadian population. The model, which includes four variables (age, sex, albuminuria, and e-GFR), gave better results than the others [19]. Although the eight-variable equation was the most accurate for predicting the risk of developing kidney failure, not all parameters are available in the initial evaluation of CKD patients; therefore, the four-variable equation may be easier to integrate into clinical practice.

In 2016, Tangri et al. published a multinational validation of the four- and eight-variable equations in 31 international cohorts, demonstrating adequate prediction of the development of kidney failure at 2 and 5 years [20, 21].

In the Colombian context, Baxter Renal Care Services has implemented a preventive program for CKD stages 3 to 5, currently serving around 20,000 patients nationwide. This program integrates the primary and specialized healthcare levels, intervening in an early and multidisciplinary for patients with CKD [22, 23].

To the best of our knowledge, the KFRE has not been validated in Colombia, and there is no scientific information to support the routine use of a CKD progression risk prediction equation that can be applied in this population. In Latin America, this risk equation has been validated recently for the Peruvian population. The authors concluded that the equation needs recalibration for this population [24]. Therefore, a validation study of the four-variable KFRE was conducted on the Colombian population.

## 2. Materials and Methods

### 2.1. Study Design and Patients

This is an external validation study of a prediction model using a retrospective multisite cohort. The population object of this study is made up of adult patients with CKD stage 3, 4, or 5 without dialysis, treated in the renal clinic network of Baxter Renal Care Services (BRCS) Colombia, in the period between January 1, 2015, and December 31, 2019, guaranteeing a follow-up of up to 2 years. The inclusion criteria were being 18 years of age or older, having a diagnosis of chronic kidney disease stage 3, 4, or 5 without dialysis (with at least two eGFR measurements according to CKD-EPI 2009 less than 60 ml/min/1.73 m^2^, in a period equal to or greater than 90 days), and having a measurement of the urine albumin-creatinine ratio (uACR). The following were excluded from the study: pregnant women, patients who had previously required dialysis or a kidney transplant, patients with an indication for dialysis without acceptance of dialysis, and patients with a follow-up period of less than two years unless the result of the dialysis initiation, transplantation, or death was presented. The study protocol was approved by the Clinical Research Ethics Committee of Renal Therapy Services Colombia (February 17, 2022, minutes, item number 002), which exempted informed consent since this study does not contain identifiable information and is a retrospective observational study.

## 3. Data Source and Analysis

### 3.1. Baseline Characteristics of the Patients

Some demographic variables, such as age, race, and sex, were included. The comorbid conditions described were a history of diabetes and cardiovascular disease (the presence of coronary artery disease and/or peripheral arterial disease). Laboratory parameters included serum creatinine and RAC.

CKD was defined and classified according to the 2012 KDIGO guidelines [7]. GFR was estimated using the CKD-EPI 2009 formula [25]. The KFRE of four variables (age, sex, baseline eGFR, and log uACR) was calculated for the non-North American population as proposed by Tangri, only on the first visit [20].

The outcomes evaluated were the need for kidney replacement therapy (hemodialysis, peritoneal dialysis, or kidney transplantation) and mortality. Data were obtained from the electronic medical records.

### 3.2. Statistical Analysis

Data are presented as the mean and standard deviation for variables with a normal distribution and as the median and interquartile range for variables with a non-normal distribution. Categorical variables are presented as frequencies and percentages. No data imputation procedure was used to control for missing data; a whole-case analysis approach was used.

Incidence rates with 95% confidence intervals were estimated for initiating kidney replacement therapy and mortality.

A Cox proportional hazards regression model was fitted, and the model's performance was assessed for discriminatory and calibration ability. Discriminative ability was set by the concordance index with Harrell's C statistic, and the time-dependent calculation of the area under the receiver operating characteristic (ROC) curve was estimated using the nearest neighbor statistical method. The optimal cut-off points for sensitivity and specificity were also estimated. Calibration was determined by the degree of agreement between the observed outcome and the probabilities predicted by the model using the Hosmer–Lemeshow statistic. Stata 16® (StataCorp. 2019. Stata statistical software: Release 16. College Station, TX: StataCorp LLC.) was used for statistical analyses.

## 4. Results

### 4.1. Patients

**Figure 1:**
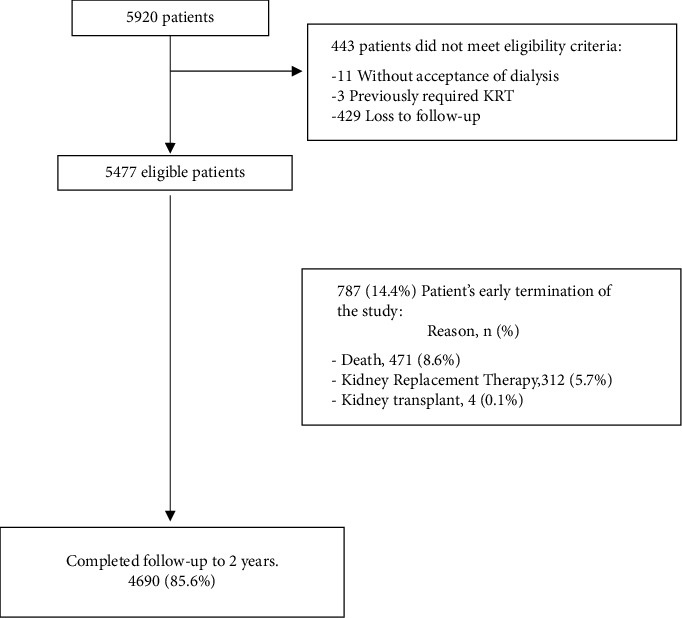
Flowchart of patients in the study. The diagram shows the flow of patients in the study. Of the 5920 patients originally included, 443 did not meet the eligibility criteria. 4690 patients completed the 2-year follow-up period.

A total of 5,477 patients were included in the analysis, 4690 (85.6%) completed the 2-year follow-up period (see Figure 1); the mean age was 72 years, the proportion of diabetic patients was 36.4%, and the mean baseline eGFR was 36 ml/min/1.73 m^2^. The dialysis initiation rate was estimated to be 3 events per 100 patient years, and the mean observation period was 683 days. The mortality rate in this population was 4.6 deaths per 100 patient years. Details are shown in Table 1.

Table 2 shows the hazard ratios (HRs) for progression to kidney failure of the four KFRE variables in the original cohort compared with the HRs in the study population, which were very similar.

### 4.2. Model Discrimination

The model's discriminative ability was evaluated using Harrell's C statistic, which was 0.88. In general, the model can adequately discriminate between patients who experience the event of interest and those who do not. See Table 3. Similarly, when discriminating by diabetes, race, and age, the C statistics of the original cohort are similar to those estimated in the Colombian study population.

**Figure 2:**
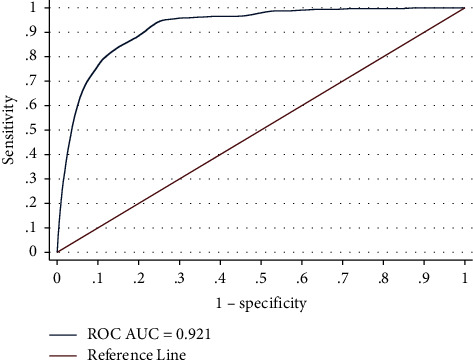
Time-dependent ROC curve. The survival function estimation method was the nearest neighbor. Sensitivity at the optimal cutpoint was 0.938, and specificity at the optimal cutpoint was 0.757.

Model discrimination was also presented graphically, with an area under the time-dependent ROC curve (AUC) of 0.92, which represents adequate discriminatory power, i.e., the AUC expresses the probability of the model to decide whether a patient has the event of interest or not. In addition, optimal points were found for sensitivity = 0.938 and specificity = 0.757. This implies that the model has a high ability to correctly identify people who will need kidney replacement therapy, achieving a remarkable 94% success rate in correctly classifying these patients. On the other hand, when considering specificity, the model demonstrates a moderate ability to correctly recognize patients who do not require KRT. Approximately 75% of patients not requiring KRT were correctly classified by the model. In predialysis care, it is fundamental to detect all cases requiring KRT. Therefore, a high sensitivity proves to be of significant value, ensuring that no potential patients for KRT are overlooked. See Figure 2.

### 4.3. Model Calibration

**Figure 3:**
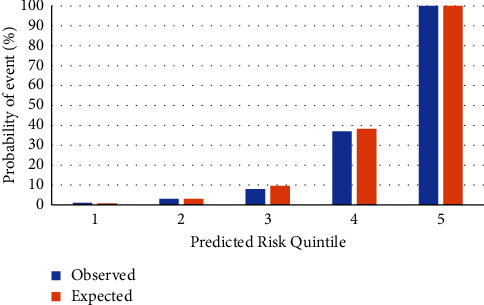
Goodness-of-fit test for the inclusion of design variables based on five quintiles of risk. Goodness-of-fit test *P* value = 0.9523.

The calibration of our model was assessed by comparing expected events with the observed events (see Figure 3) using the Hosmer-Lemeshow statistic; the goodness of fit test was 0.952, reflecting the agreement between the estimated predictions and the observed events.

## 5. Discussion

In modern healthcare systems, there is a growing interest in developing predictive tools for outcomes of interest for the population. This is the case with CKD and predicting the risk of progression. In this direction, Tangri et al. have developed a risk equation for kidney failure, aligning the risk estimate with the availability of resources in a health system, thus personalizing the care of patients with CKD [26]. As a derived risk prediction model moves from one population to another, it is essential to assess whether the model being validated adequately predicts risk in this new population so that its results can be generalized to similar individuals, even if they differ in many ways from those from which the model was derived [21].

The present study of external validation of the four-variable KFRE in the Colombian population has revealed a remarkable discriminatory capacity for this model, both for the C statistic and the area under the curve consistently being around 0.9, indicating an excellent discriminative ability, very similar to that reported in the meta-analysis for the multinational evaluation of this risk equation [21]. It should be noted that the proportion of patients with micro- or macroalbuminuria in our population is slightly higher than that found in the derivation cohort [20] and in the summary measure of the multinational evaluation with meta-analysis [21]. However, it is also interesting to note that this proportion of patients with albuminuria is very variable in the various studies included in this meta-analysis, an aspect that in our view confers relevance to the validation and does not invalidate the results. Otherwise, in Latin America, Bravo *et* al. found that the equation has good discrimination but poor calibration in the Peruvian population; that is, the model underestimates the risk of kidney failure in the short term and overestimates it in the long term; these results could be related mainly to the way the population in the study was selected [24].

On the other hand, the calibration of the model yielded a goodness-of-fit test more significant than 0.9, with graphical evidence of excellent calibration in all risk quintiles, a finding somewhat different from that reported in the referenced meta-analysis for non-North American cohorts [21], where there was a tendency to overestimate risk. Consequently, the two-year predictive performance of the four-variable KFRE in Colombian patients with stage 3, 4, or 5 CKD without the need for dialysis could be considered quite adequate.

In discussing the limitations of our study, it is worth noting that patients who were candidates for dialysis but opted for conservative treatment and refused therapy were excluded, although this number was relatively marginal. Furthermore, in the present study, the death event was censored and not considered a competing risk with the initiation of kidney replacement therapy (KRT). It is important to note that some authors suggest treating death as a competing risk only in predictions with a longer follow-up, such as five years, to avoid biased estimates [27].

In 2021, Ramspek et al. performed an external validation of the kidney failure equation that included death as a risk competing with the need for KRT. This study found that models with a short prediction horizon (one or two years) had similar results to models that ignored competing risks. However, models that predict the 5-year risk and do not include death as a competing risk were found to overestimate the risk of progression to kidney failure [28]. To predict the risk of kidney failure in the short term (two years), we, like other authors, recommend the use of a four-variable KFRE without adjustments for competing risks [28].

Implementing this tool in clinical practice will enable risk-based care and optimize outcomes in the CKD population. Moreover, it can also identify groups at high risk of progression, inform public health decisions, and enhance the cost-effectiveness of care for CKD patients.

## 6. Conclusions

In this Colombian cohort, the four-variable KFRE with a two-year prediction horizon has excellent calibration and discrimination, and its use in the care of CKD Colombian patients is recommended.

## Figures and Tables

**Table 1:** Baseline demographic and clinical characteristics.

Characteristics	Predialysis program *N* = 5477 *n* (%)
Demographics	
Age, mean (SD), years	72.0 (13.4)
<65 years	1323 (24.2)
≥65 years	4154 (75.8)
Sex, *n* (%)	
Male	2840 (51.8)
Female	2637 (48.2)
Ethnicity, *n* (%)	
Others	5439 (99.3)
Afro-American	38 (0.7)
Comorbid conditions	
Diabetes	1993 (36.4)
Cardiovascular disease,^a^	799 (14.6)
Laboratory data	
GFR, mL/min/1.73 m^2^ baseline, mean (SD)	36.1 (13.6)
30–59	3523 (64.3)
15–29	1659 (30.3)
<15	295 (5.4)
Serum creatinine, mean (SD), mg/dL	1.9 (0.8)
Urine albumin-to-creatinine ratio, mg/g, median (IQR)	112 (30, 496)
<30	1325 (24.2)
30–299	2331 (42.6)
≥300	1821 (33.2)
Outcomes	
Observation time, days	683 (132.5)
Kidney failure events	
Hemodialysis	101
Peritoneal dialysis	211
Kidney transplantation	4
Mortality rate per 100 person-year	4.6 [4.2–5.0]
KRT rate per 100 person-year	3.0 [2.9–3.6]

GFR, glomerular filtration rate; IQR, interquartile range; SD, standard deviation. ^a^Cardio-vascular disease is defined as the presence of coronary artery disease or peripheral vascular disease. KRT: kidney replacement therapy.

**Table 2:** Hazard ratios for kidney failure of the component variables in the original vs. external cohort 4 variable equation.

4-Variable equation	Hazard ratio (95% CI) original	Hazard ratio (95% CI) external validation
Age per 1-0 years older	0.80 (0.75–0.86)	0.74 (0.69–0.79)
Male sex	1.26 (1.04–1.58)	1.27 (1.02–1.59)
eGFR per 5 mL/min/1.73 m^2^	0.57 (0.54–0.61)	0.55 (0.51–0.59)
uACR per log increase	1.60 (1.44–1.71)	1.64 (1.50–1.79)

CI, confidence interval; uACR, urinary albumin-creatinine ratio; eGFR, estimated glomerular filtration rate.

**Table 3:** Discrimination: 2-year predicted probability of kidney failure.

Cohort patients	C statistic (95% CI) original	C statistic (95% CI) external validation
*Diabetes*		
Yes	0.90	0.88
No	0.92	0.92

*Black*		
Yes	0.91	0.92
No	0.90	0.91

*Age, years*		
≥65	0.90	0.93
<65	0.90	0.85

CI, confidence interval; Harrell C statistic.

## Data Availability

The security of the database is consistent with the confidentiality requirements that protect patient privacy as part of the study protocol. Data availability is restricted to ensure patient privacy. The full study protocol and database are available upon request from the principal investigator (carolina_larrarte@baxter.com).
